# ﻿Three new species of *Boesenbergia* (Zingiberaceae) from Sabah, Malaysia

**DOI:** 10.3897/phytokeys.247.107961

**Published:** 2024-10-08

**Authors:** Nyee Fan Lam, Halijah Ibrahim, Yen Yen Sam, Rozainah Mohammad Zakaria, Axel Dalberg Poulsen

**Affiliations:** 1 Faculty of Science, University of Malaya, Jalan Professor Diraja Ungku Aziz, 50603, Kuala Lumpur, Malaysia University of Malaya Kuala Lumpur Malaysia; 2 Institute for Tropical Biology and Conservation, Universiti Malaysia Sabah, Jalan UMS, 88400 Kota Kinabalu, Malaysia Universiti Malaysia Sabah Kota Kinabalu Malaysia; 3 Forest Research Institute Malaysia, Jalan FRIM, 52109 Kepong, Malaysia Forest Research Institute Malaysia Kepong Malaysia; 4 Royal Botanic Garden, Science, 20A Inverleith Row, Edinburgh EH35LR, Scotland, UK Royal Botanic Garden Edinburgh United Kingdom

**Keywords:** Biodiversity, Borneo, endemic, new species, wild gingers

## Abstract

Three new species of *Boesenbergia*, *B.bosuangii***sp. nov.**, *B.ganaensis***sp. nov.** and *B.gokusingii***sp. nov.** were discovered in Sabah, Malaysia. *Boesenbergiabosuangii* is similar to *B.stenophylla* R.M.Sm. in the narrowly ovate lamina but differs in the shape of the bract and the calyx. *Boesenbergiaganaensis* is closely allied to *B.burttiana* R.M.Sm. but differs in the absence of a ligule, the longer petiole, the obtuse leaf base, the acute leaf apex, the bilobed calyx and the anther dehiscing by pores. Finally, *B.gokusingii* is similar to *B.variegata* R.M.Sm., by the single leafy shoot but differs in having an unequal, ovate lamina, a cordate leaf base, an acute leaf apex and the anther dehiscing by pores. The three new species are described and illustrated in detail. With the addition of these new species, there are in total 13 species with one variety in Sabah.

## ﻿Introduction

The genus *Boesenbergia* was initially classified in the tribe Hedychieae in the family Zingiberaceae (Burtt and Smith 1972). Considering molecular data, *Boesenbergia* was subsequently placed in the tribe Zingibereae, subfamily Zingiberoideae (Kress et al. 2002). Eight species from the genus *Haplochorema* and five species from the genus *Caulokaempferia* were added to the genus *Boesenbergia* (Mood et al. 2014; Mood et al. 2020) and there are currently 99 species of *Boesenbergia* (Lam et al. 2022).

The character, which distinguishes *Boesenbergia* from all other Zingiberoideae genera, is that the first flowers appear at the top of the inflorescence and flowering progresses towards the base (Poulsen and Searle 2005; Sakai and Nagamasu 2006, 2009; Mood et al. 2020). The flowers are usually white or pale yellow or orange with a spoon-shaped labellum ornamented with red and/or pink. The diagnostic characters of *Boesenbergia* in Sabah are the growth form (creeping or erect), number of leave per shoot (1 to many), anther dehiscence (slits or pores), length of petiole, and shape of the lamina, including base and apex (Lam 2023).

Borneo harbours approximately 38 species of *Boesenbergia* (Smith 1987; Ibrahim 1992; Sirirugsa 1992; Poulsen 1993; Larsen 1997; Cowley 1998, 2000; Larsen et al. 1999; Saensouk and Larsen 2001; Sakai and Nagamasu 2006, 2009; Lamb et al. 2013; Lam et al. 2022) of which only 10 species and one variety have been reported in Sabah (Lam et al. 2022). The distribution pattern of the species within Sabah is poorly known but they seem to be usually found in riverine, limestone, and near waterfalls in primary forests. Therefore, this study is focused on revealing the species diversity of *Boesenbergia* in Sabah.

## ﻿Materials and methods

Field collections were made between August 2016 and August 2017 at Ranau and Kimanis Districts. The morphology of the new species was analyzed using living plants with reference to herbarium materials (E, K, KEP, KUL, SAN and SING). The procedures of the fieldwork and measurements were conducted based on Lam et al. (2022).

Information collected during fieldwork and herbaria was recorded in the Taxon Data Information Sheet (TDIS) form. The form consist of five sections, namely, taxon attributes, geographic range and demographic details on population (Chua et al. 2010). Ground points of collections were used in the IUCN Red List assessments (IUCN 2022). The assessments of Extent of occurrence (EOO) and Area of occupancy (AOO) and maps were plotted with GeoCAT (Bachman et al. 2011).

### ﻿Key to the species of *Boesenbergia* in Borneo (modified from Sakai and Nagamasu 2009)

**Table:** 

1	Creeping; leafy shoots normally single-leaved; inflorescence more or less sessile; anther dehiscing by slits	**2**
–	Erect; leafy shoots with one to many leaves; inflorescence sessile or long pedunculate; anther dehiscing by slits or pores	**7**
2	Lamina more or less circular, obtuse or obscurely emarginate at apex	***B.orbiculata***
–	Lamina elliptic or narrowly ovate, acute at apex	**3**
3	Lamina plain green	**4**
–	Lamina variegated	**5**
4	Floral tube pubescent outside; flower not red at throat; labellum entire	***B.flavoalba***
–	Floral tube glabrous outside; flower red at throat labellum bilobed	***B.flavorubra***
5	Petiole 2–3 cm long; lamina 7–12 by 2.5–7 cm, dark green with a band of lighter green up the midrib, variegation sometimes extending to the main lateral veins	***B.variegata***
–	Petiole < 0.5 cm long; lamina shorter than 9 cm with width less than 5.5 cm	**6**
6	Lamina 4–8 by 1.5–2 cm, mid-green with a broad silver band on either side of the midrib above, surface glabrous	***B.kerbyi***
–	Lamina 8.6 by 5.5 cm, upper surface undulating from green to dark green	***B.gokusingii***
7	Fertile shoot single-leaved, rarely bladeless or 2- or 3-leaved	**8**
–	Fertile shoot with two or more leaves	**12**
8	Lamina 50 by 12 cm or larger	***B.grandifolia***
–	Lamina much smaller, not exceeding 30 cm long	**9**
9	Base of the lamina deeply cordate	***B.cordata***
–	Base of the lamina ± attenutate	**10**
10	Petiole 17–34 cm long	***B.bruneiana***
–	Petiole not exceeding 17 cm	**11**
11	Lamina 7–12 cm wide; petiole robust ca. 5 mm thick; lamina with appressed hairs especially around midrib below	***B.lambirensis***
–	Lamina less than 7 cm wide; petiole slender, 2 mm or less thick; leaves glabrous	***B.ischonosiphon***
12	Lamina large, much longer than 30 cm	**13**
–	Lamina shorter than 30 cm, if longer narrower than 7 cm	**17**
13	Leaf base thickened with outermost bracts forming a bucket or vase-like structure enclosing inflorescence sometimes together with sheaths of upper leaves; petiole 42–50 cm long	***B.jangaruni***
–	Leaf base or sheaths not thickened as above, long-attenuate forming a winged petiole less than 25 cm long	**14**
14	Anther dehiscing by subapical pores or slits	**15**
–	Anther dehiscing by pores	**16**
15	Inflorescence densely pubescent; anther ca. 3 mm long, dehiscing by subapical pores	***B.hosensis***
–	Inflorescence glabrous, anther ca. 10 mm long, dehiscing by longitudinal slits ca. 6 mm long	***B.armeniaca***
16	Leaf sheath sparsely hairy or glabrous; bracts 5–8 cm; floral tube 8–10 cm; ovary glabrous	***B.grandis***
–	Leaf sheath densely hairy; bracts 2–3.5 cm long; floral tube ca. 5.5 cm long; ovary densely hairy in upper half	***B.lysichitoides***
17	Inflorescence long exserted from the leaf sheaths when fully grown, spindle-shaped; flowers red and white	***B.pulchella***
–	Inflorescence never long exserted or spindle-shaped; flower colours various	**18**
18	Leaves linear; arrangement of blades strongly flabellate	**19**
–	Leaves elliptic, narrowly ovate or rarely linear to narrowly ovate; arrangement of blades never flabellate	**20**
19	Flower plain yellow; anther dehiscing by apical pores; bracts 3.5–6.5 cm	***B.flabellata***
–	Flower white, yellow in the centre, pink at the base; anther dehiscing by slits; bracts < 3 cm	***B.burttiana***
20	Leaves variegated	**21**
–	Leaves plain green	**25**
21	Leaves bullate, dark green around main veins and almost silvery on raised area	***B.hutchinsonii***
–	Leaves smooth with a silverish or light green central cloud	**22**
22	Petiole never exceeding 3 cm, lamina narrowly obovate with attenuate base	***B.hirta***
–	Petiole usually much longer than 3 cm, lamina narrowly ovate to elliptic with cuneate base	**23**
23	Leaves with a silvery cloud; flowers yellow, labellum orange-spotted	***B.ornata***
–	Leaves with yellow central cloud; flowers orange or white with some yellow and reddish purple	**24**
24	Leaves 5–12 by 3–4 cm; flower orange, darker at base of labellum; anther dehiscing throughout its entire length	***B.aurantiaca***
–	Leaves 18–23 by 4–6 cm flower white with some yellow and reddish purple; anther dehiscing by apical pores, or anther dehiscent only in upper 2/3	***B.belalongensis***
25	At least a few uppermost leaf sheaths thickened and forming a cup-shaped structure	**26**
–	Leaf sheath not thickened as above	**27**
26	Innermost leaf sheaths enclosing inflorescence much shorter and wider than outer ones; leaves drying darkish brown	***B.laevivaginata***
–	All leaves with more or less equal laminae; leaves green or grey-green when dry	***B.urceoligena***
27	Anther dehiscing by slits throughout their length	**28**
–	Anther dehiscing by pores	32
28	Petiole < 8 cm	***B.imbakensis***
–	Petiole > 10 cm	**29**
29	Calyx unilaterally incised (split on one side)	**30**
–	Calyx tubular	***B.sugudensis***
30	Lamina wider than 4 cm	**31**
–	Lamina less than 4 cm wide	**33**
31	Petiole up to 4 cm long; lamina longer than 10 cm	**32**
–	Petiole to 2 cm long; lamina 5.2–6.5 by 3.4–3.6 cm	***B.truncata***
32	Lamina 13–16 cm long	***B.apiculata***
–	Lamina at least 11.5 by 5.5 cm	***B.ganaensis***
33	Lamina narrowly ovate, more than 12 cm	**34**
–	Lamina slightly ovate much shorter, up to 12 cm long, if longer, petiole much shorter than 7 cm	**35**
34	Lamina 12–20 by 1.5–3 cm; petiole usually to 7–8 cm	***B.stenophylla***
–	Lamina 19 by 3 cm; petiole usually at least 10.5 cm	***B.bosuangii***
35	Leaf sheath and ligule hirsute	***B.parva***
–	Leaf sheath and ligule almost glabrous	**36**
36	Flower yellow-orange	***B.oligosperma***
–	Flower white and yellow, occasionally red in throat	***B.subulata***

## ﻿Taxonomy

### ﻿ Boesenbergia bosuangii

Taxon classificationPlantae

N.F.Lam sp. nov.

069BD6E3-BB80-535F-A977-A815C7CE5B34

urn:lsid:ipni.org:names:77349804-1

Figs 1 , 2

#### Diagnosis.

The new species resembles *B.stenophylla* in having a narrowly ovate lamina, but differs in the shape of the bract (linear elliptic vs. cymbiform) and a tubular calyx (vs. tridentate) (Table 1).

**Table 1.** Distinguishing morphological characters of *B.bosuangii* and *B.stenophylla*.

Characters	*B.bosuangii*	*B.stenophylla*
Plant height	to 30 cm	To about 42 cm
Petiole	7.5 cm	10–18 cm
Leaf apex	Acuminate	Slightly acuminate
Bracts	2.7 cm, linear elliptic, pubescent	4 × 4.5 cm, cymbiform, glabrous
Calyx	2-lobed, pubescent	3-lobed, glabrous
Labellum	Yellow band from base in the centre spreading towards apex, faint red at base and dark red bands bordering the yellow band, obovate, 1.2 × 0.8 cm	White with yellow centre, flabelliform, 2.5 × 2 cm
Lateral corolla lobe	White, 1 × 0.25 cm	Pale yellow, 1.5 × 0.4 cm,

**Figure 1.**
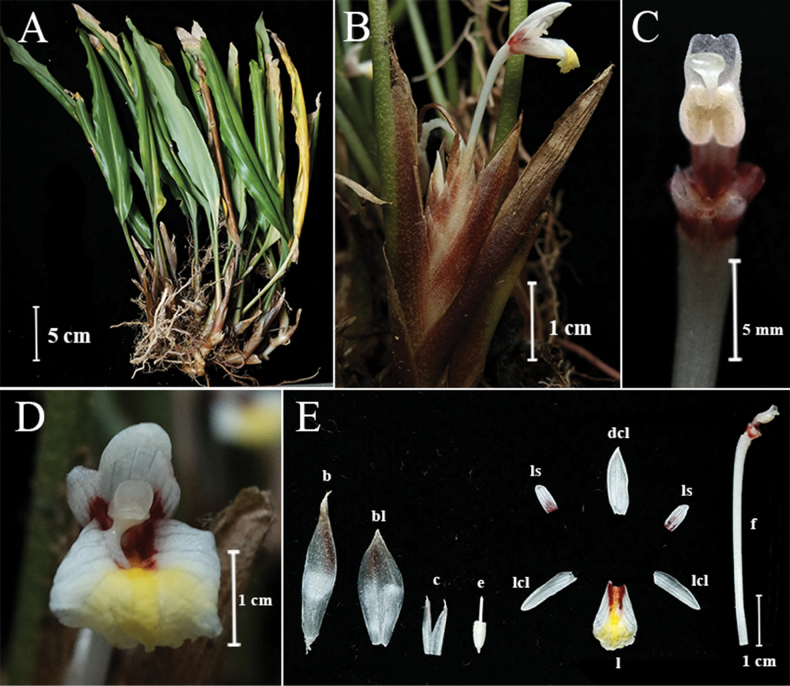
*Boesenbergiabosuangii***A** habit **B** spike with one open flower **C** stamen, ventral view **D** flower **E** Bract, bracteole, calyx, epigynous gland, corolla lobes, staminodes, labellum, floral tube with stamen. (Photograph of Lam Nyee Fan 337; Photos: Lam Nyee Fan).

#### Type.

Malaysia. Borneo. Sabah. Cultivated at Kipandi Park, Moyog, 05°54.68'N, 116°06.27'E, 700 m elevation, 8 August 2016, *Lam Nyee Fan 356* (holotype BORH!, isotype SAN). Original material collected by Linus Gokusing (BS-23) at Marakau, Gana-gana, Ranau, Sabah, 06°12.24'N, 116°46.03'E, 480–500 m elevation, 3 August 2010.

**Figure 2.**
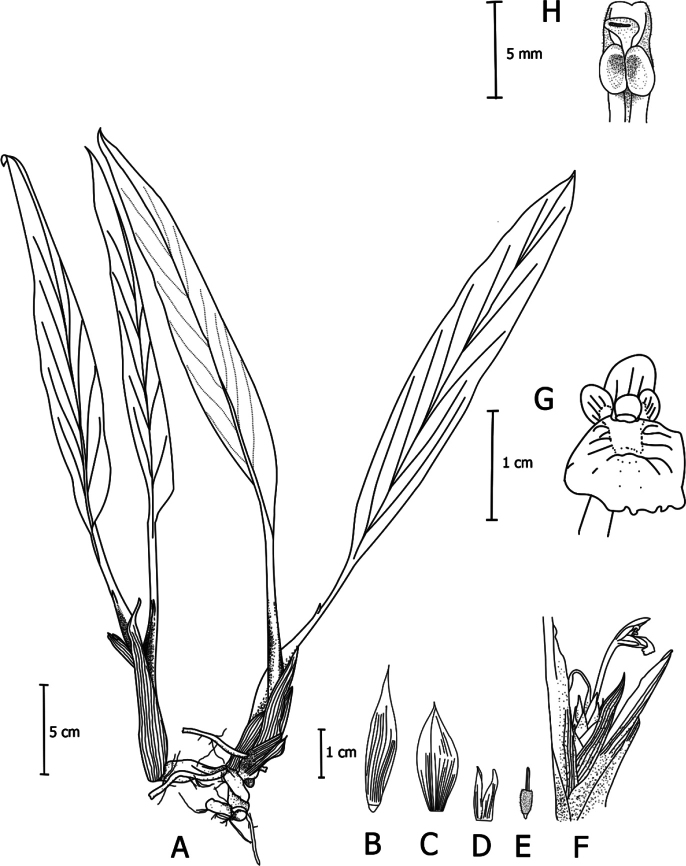
*Boesenbergiabosuangii* Lam N.F., sp. nov. **A** habit **B** bract **C** bracteole **D** calyx **E** epigynous glands, **F** spike with one open flower **G** flower **H** stamen, ventral view (Drawing by Lam Nyee Fan). Scale bars: 5 cm (**A**); 1 cm (**B, C, D, E, F, G**); 5 mm (**H**).

#### Description.

Terrestrial, evergreen, herb. ***Rhizome*** fibrous, subterranean, ca. 1 cm in diameter with 7 cm internodes, white to light brown, base ca. 0.6 cm in diameter, roots white, up to 18 cm long. ***Leafy shoots*** 29.5 cm tall, with 1–2 leaves, with 2–3 outer leafless sheaths, 6.0–6.5 × 0.7–1.1 cm, purple brownish, glabrous. ***Ligule*** ca. 0.55 cm long, caudate, brown, glabrous. ***Petiole*** 6–7.5 cm long, canaliculate, green, reddish at base. ***Lamina*** narrowly ovate, 17–17.4 × 2.5–2.75 cm, erect, dark green above, pale green beneath, glabrous, base attenuate, margin entire, apex acuminate, with acumen ca. 2 mm. ***Inflorescence*** ca. 2.8 cm, peduncle 1–2 cm, with up to 6 flowers arranged in a one-sided spiral, one flower open at a time. ***Fertile bracts*** linear elliptic, ca. 2.7 cm long, white, reddish at apex, pubescent, margin entire, apex attenuate. ***Bracteole*** elliptic, ca. 2 × 0.75 cm, translucent, pubescent, margin entire, apex acute. ***Flower*** white, born singly from each bract; calyx 1 cm long, tubular, 2-lobed, translucent, pubescent on both surfaces; corolla tube ca. 3.6 cm long, ca. 1.5 mm wide at base, lobes white, glabrous throughout, dorsal lobe ovate-oblong, ca. 1.1 × 0.5 cm, linear elliptic, concave, white, glabrous, apex acute, lateral lobes ovate, ca. 1.0 × 0.25 cm, oblong, glabrous, apex rounded; labellum, obovate, ca. 1.2 cm × 0.8 cm curved-backward, longer than corolla lobes, with yellow band from base in the centre spreading towards lip, faint red at base and dark red bands on both sides, glabrous; lateral staminodes white, reddish at base, linear, ca. 0.6 × 0.25 cm, glabrous; stamen white throughout, ca. 0.45 cm long, filament ca. 4 mm × 1 mm (widest at base), pubescent, anther ca. 0.5 × 0.3 cm, glabrous, anther crest ca. 0.5 × 2 mm, bilobed, pubescent, thecae oblong, ca. 0.3 × 0.1 cm, glabrous, dehiscing by pores; ovary ca. 4 × 1.5 mm, stigma cup-shaped, glabrous; epigynous glands, two, ca. 0.45 cm long, linear, apex truncate, white. ***Fruit*** not seen.

#### Distribution.

Endemic to Borneo, Sabah.

#### Other specimens seen.

Malaysia. Sabah. Telupid District, Taviu Forest Reserves, c. 200 m elevation, lowland forest, 17 May 2001, Sundaling D, SAN142970 (SING!, SAN!).

#### Etymology.

The species is named after Dr. Steven Bosuang, owner of Kipandi Park. He is an entomologist doing conservation efforts on insects and plants of Sabah. His collaborations with local and overseas scientist produced many research papers and reports for the conservation of Sabah.

#### Ecology.

Riverine area in mixed dipterocarp forest, 50–300 m elevation, flowering in August.

#### Conservation status.

**Figure 3.**
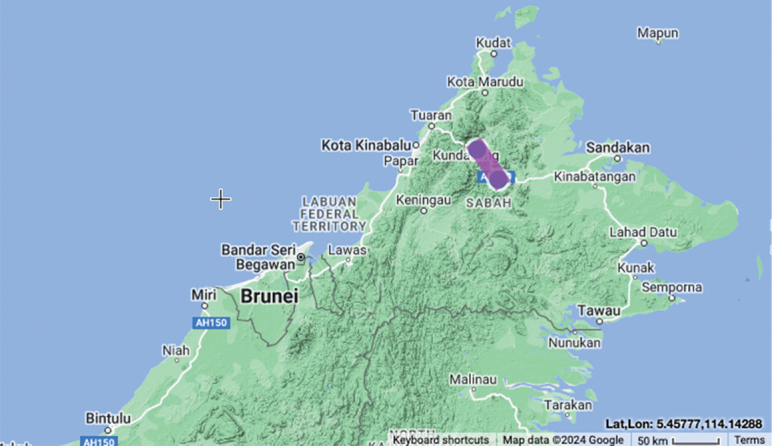
Distribution map of *Boesenbergiabosuangii*, EOO = 0 km^2^, AOO = 8 km^2^ (Bachman et al. 2011).

Vulnerable VU D1. *Boesenbergiabosuangii* is endemic to Sabah. This species is found at Ranau and Telupid Districts. Due to the small, restricted populations outside protected area and possible threats from development, landslides and flooding, this species is assessed as VU (Fig. 3).

### ﻿ Boesenbergia ganaensis

Taxon classificationPlantae

N.F.Lam sp. nov.

3C0CBD2F-CA10-5CE9-B9B9-47DD94B2A05E

urn:lsid:ipni.org:names:77349805-1

Figs 4 , 5

#### Diagnosis.

The new species resembles *B.burttiana* by having a narrowly ovate lamina and a similar plant height, but differs in having an obtuse leaf base, an acute leaf apex (vs. attenuate leaf base, slightly acuminate leaf apex), absence of ligule, a longer petiole (4 cm vs.1.8 cm) and the anther thecae dehiscing by pores (vs. slit) (Table 2).

**Table 2.** Distinguishing morphological characters of *B.ganaensis* and *B.burttiana*.

Characters	*B.ganaensis*	*B.burttiana*
Ligule	Absent	0.55 cm, acuminate, reddish, glabrous
Petiole	4 cm, green, grooved	1.0–1.8 cm, green, without winged
Lamina	Upper surface dark green	Upper surface light green
Leaf size	11.5 × 5.5 cm	12–20 × 1.5–3 cm
Leaf base	Obtuse	Attenuate
Leaf apex	Acute	Slightly acuminate
Bracts	2.4 cm long, white, narrowly ovate, almost translucent	4 cm long, cymbiform
Labellum	Yellow band from base in the middle, spreading to almost entire surface near apex with lighter yellow laterally, red bands from 2/3 of the centre yellow band towards side, 1.1 × 0.8 cm	White, with yellow in the centre, pink at the base, 2.5 × 2 cm
Lateral corolla lobe	Glabrous, white, 1.2 × 0.2 cm, emarginated, white with link pink ad yellowish at base, light pink patches towards lip	Slightly pubescent, pale yellow, 1.5 × 0.8 cm
Anther	reddish, pubescent	White, glabrous
Anther dehiscence	Pore	Slit
Stigma	Truncate	Rounded

**Figure 4.**
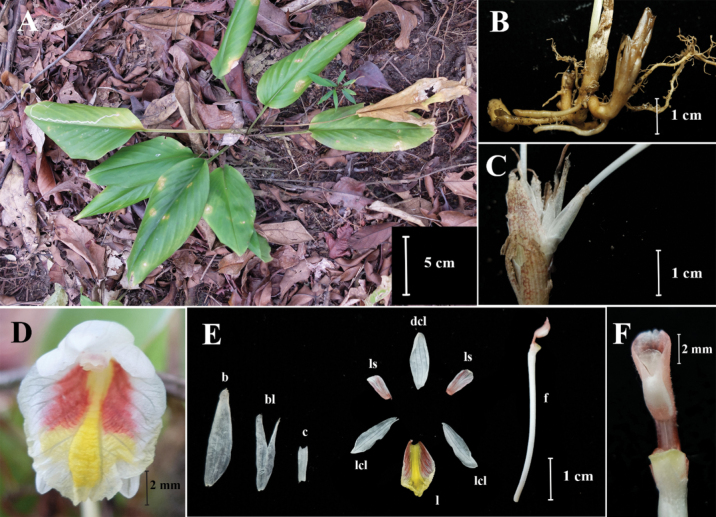
*Boesenbergiaganaensis***A** habit **B** rhizome and roots **C** spike with one open flower **D** flower **E** bract, bracteole, calyx, corolla lobes, staminodes, labellum, floral tube with stamen **F** stamen, ventral view (Photos: Lam Nyee Fan).

#### Type.

Malaysia. Borneo. Sabah. Cultivated at Kipandi Park, Moyog, 05°54.68'N, 116°06.27'E, 700 m elevation. 8 Aug 2016, *Lam Nyee Fan 348* (holotype BORH!, isotype SAN). Original material collected from Ranau, Gana-gana, by Linus Gokusing (BS-15), 05°53.16'N, 116°39.30'E, 700 m elevation, 2 February 2013.

**Figure 5.**
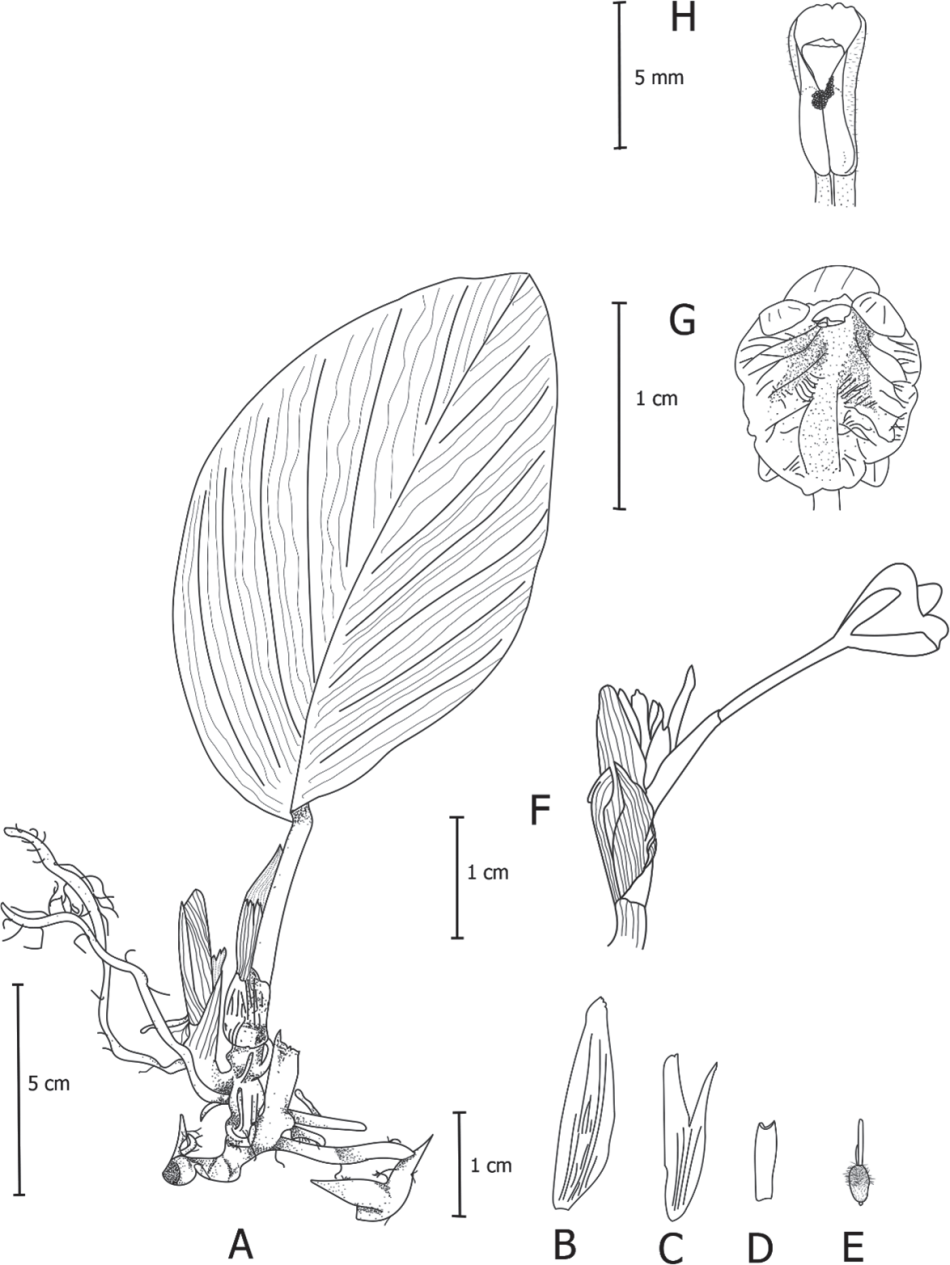
*Boesenbergiaganaensis* Lam N.F., sp. nov. **A** habit, lateral view **B** bract **C** bracteole **D** calyx **E** epigynous glands **F** spike with one open flower **G** flower **H** stamen, ventral view (Drawing by Lam Nyee Fan). Scale bars: 5 cm (**A**); 1 cm (**B, C, D, E, F, G**); 1 mm (**H**).

#### Description.

Terrestrial, evergreen, herb. ***Rhizome*** fibrous, subterranean, base ca. 0.4 cm in diameter, light brown, roots white, ca. 4 cm long. ***Leafy shoots*** ca. 14 cm tall, with 2–4 sheaths, ca. 3.5 × 2.5 cm, glabrous, green, margins entire. ***Ligule*** absent. ***Petiole*** 2.8–4 cm long, grooved, green. Leafy shoots 1–2. ***Lamina*** elliptic, 10–11.5 × 4–5.5 cm, dark green above, green beneath, glabrous, margin entire; base obtuse, apex acute with acumen ca. 1 mm. ***Inflorescence*** ca. 2.7 × 0.4 cm, peduncle ca. 0.35 cm with up to 10 flowers arranged in a one-sided spiral, one flower open at a time. ***Fertile bracts*** narrowly ovate, ca. 2.4 × 0.5 cm, white, outer and inner surfaces glabrous, almost translucent, margin entire, apex acute. ***Bracteole*** elliptic, ca. 1.8 × 0.5 cm, white, outer and inner surfaces glabrous, translucent, margin entire, apex acute. ***Flower*** white, born singly from each bract, calyx 0.7 cm long, tubular, white, glabrous, corolla tube white, glabrous, apex rounded, dorsal lobe elliptic, ca. 1.3 × 0.35 cm, concave, lateral lobes elliptic, ca. 1.2 × 0.2 cm, labellum bucket shaped, obovate (when flattened), ca. 1.1 cm × 0.8 cm, yellow band from base in the middle, spreading to almost entire surface towards the apex with lighter yellow laterally with red bands from 2/3 of the centre yellow band towards side, lateral staminodes oblong, ca. 0.7 × 0.2 cm, white, apex acute, glabrous, stamen ca. 1.2 cm long; filament ca. 7 × 1 mm (widest at base), pubescent adaxially and abaxially, anther ca. 3.5 mm long, pubescent; anther crest trilobed, pubescent; thecae oblong, ca. 0.3 × 0.1 cm, white, glabrous, dehiscing by pores, stigma truncate apex, white, glabrous, epigynous glands 0.2–0.4 cm long, linear, apex pointed. ***Fruit*** not seen.

#### Distribution.

Endemic in Borneo, Sabah; known only from the type locality at Kampung [village] Gana-gana, Ranau.

#### Etymology.

The species epithet refers to the location where the species was collected.

#### Ecology.

Granite area at 500–600 m elevation.

#### Conservation status.

**Figure 6.**
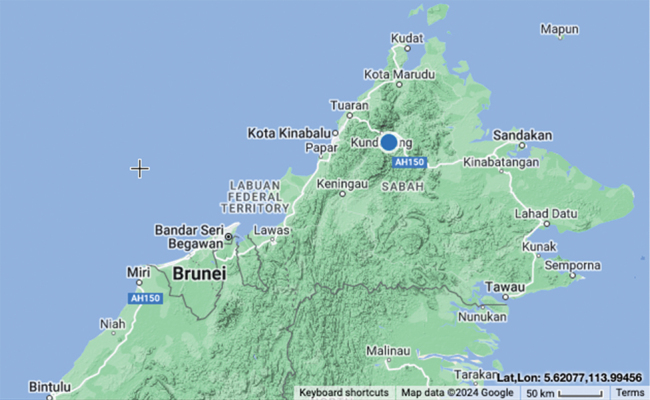
Distribution map of *Boesenbergiaganaensis*, EOO = 0 km^2^, AOO = 4 km^2^ (Bachman et al. 2011).

Vulnerable (VU D2). The species is endemic to Sabah and only found at Ranau, Sabah, Malaysia. There were only four populations found at the site of collection and it has not been found outside the type locality (Fig. 6).

### ﻿ Boesenbergia gokusingii

Taxon classificationPlantae

N.F.Lam sp. nov.

67185761-EDD4-5CEB-8EE6-F0DEA2E17C58

urn:lsid:ipni.org:names:77349806-1

Figs 7 , 8

#### Diagnosis.

The new species resembles *B.variegata* R.M.Sm. by having single-leaved shoots and a short petiole, but differs in having unequal ovate lamina (vs. elliptic), a cordate leaf base (vs. rounded or subcordate), an acute leaf apex (vs. subacute) and the anther thecae dehiscing by pores (vs. slits) (Table 3).

**Table 3.** Distinguishing morphological characters of *B.gokusingii* and *B.variegata*.

Characters	*B.gokusingii*	*B.variegata*
Ligule length	1 mm	2–3 mm
Lamina	Unequal ovate, upper surface undulating ranging from green to dark green	Elliptic, upper surface dark green with a lighter band along the midrib, sometimes variegated variegation extends to the lateral veins
Leaf size (cm)	8.6 × 5.5 cm	7–12 × 2.5–7 cm
Leaf base	Cordate	Rounded or subcordate
Leaf apex	Acute	Subacute
Bracts	1.6 cm long, linear to narrowly ovate, glabrous	0.6–0.8 cm long, ovate, acute, pubescent
Labellum	With light yellow at base, darker yellow spreading towards lip, obovate, curved-forward, 0.7 × 0.4 cm	Cream with a deep yellow spot in the centre and a deep red spot at the base, elliptic, 1.0 × 0.7 cm
Lateral corolla lobe	Glabrous, 0.7 cm long	Densely pubescent, 2 cm long
Anther dehiscence	Pore	Slit

**Figure 7.**
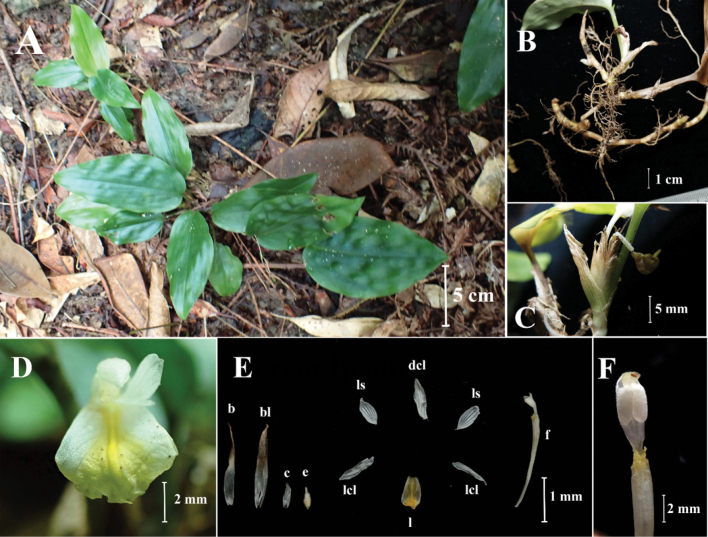
*Boesenbergiagokusingii***A** habit **B** rhizome and roots **C** spike with one open flower **D** flower **E** bract, bracteole, calyx, corolla lobes, staminodes, labellum, floral tube with stamen **F** stamen, ventral view (Photos: Lam Nyee Fan).

#### Type.

Malaysia. Borneo. Sabah. Cultivated at Kipandi Park, Moyog, 05°54.68'N, 116°06.27'E, 700 m elevation. 8 Aug 2016, *Lam Nyee Fan 361* (holotype BORH!, isotype SAN). Original material collected from Tawau by Linus Gokusing (BS-21b), 05°11.93'N, 117°25.40'E, 500–600 m elevation, 1 Aug 2015.

**Figure 8.**
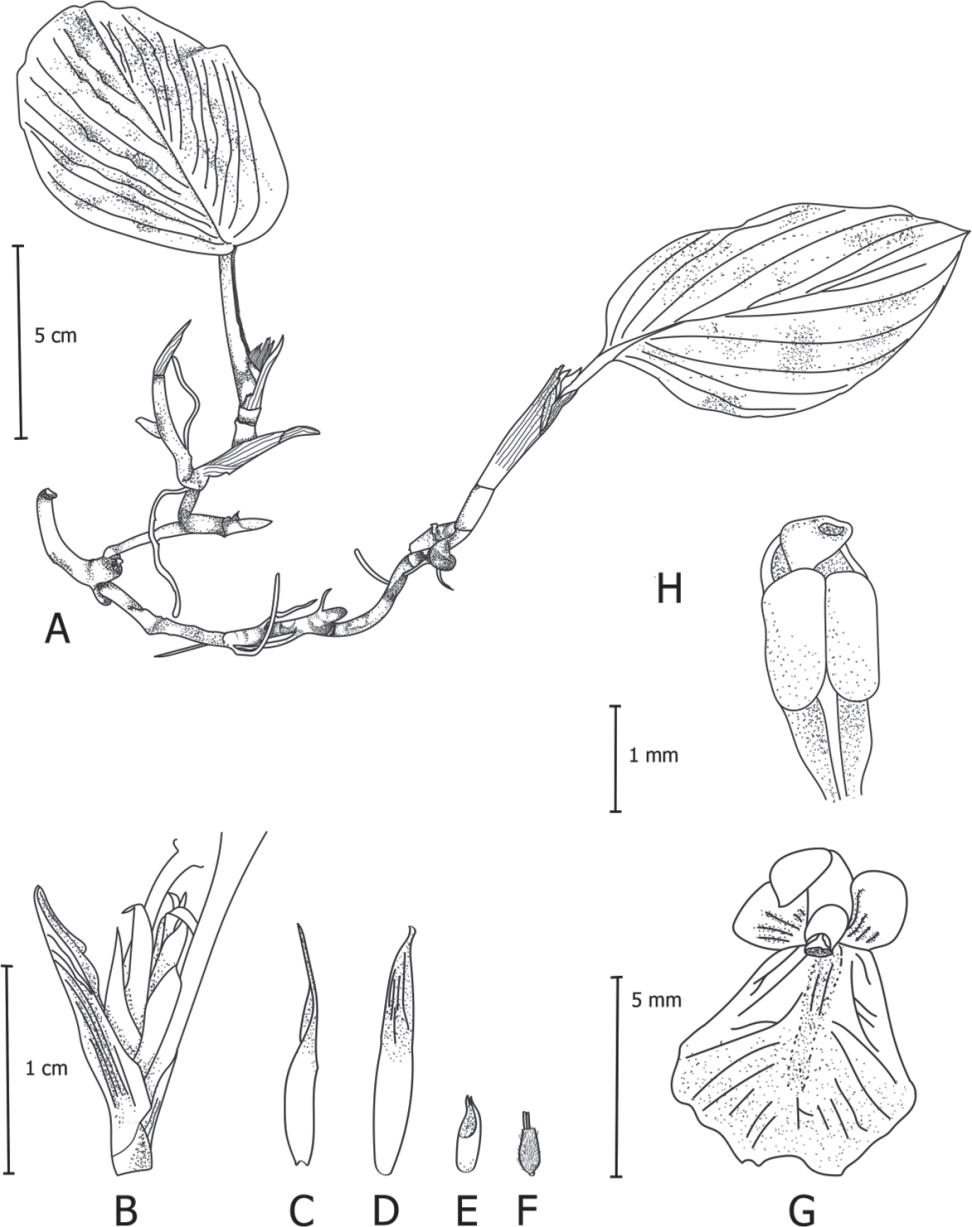
*Boesenbergiagokusingii* Lam N.F., sp. nov. **A** habit, lateral view **B** spike **C** bract **D** bracteole **E** calyx **F** epigynous glands **G** flower **H** stamen, ventral view (Drawing by Lam Nyee Fan). Scale bars: 5 cm (**A**); 1 cm (**B, C, D, E, F**); 5 mm (**G**); 1 mm (**H**).

#### Description.

Terrestrial, evergreen, herb. ***Rhizome*** fibrous, subterranean, base ca. 0.4 cm in diameter, roots brown. ***Leafy shoots*** ca. 8.5 cm tall, with 1–2 sheaths, ca. 2 × 1.5 cm, glabrous, green, margins entire. ***Ligule*** 1 mm long, acute, light brown, glabrous. ***Petiole*** 2.8–3.5 cm long, grooved, green. Leafy shoots single. ***Lamina*** unequal ovate, 7.5–8.6 × 5–5.5 cm, upper surface undulating ranging from green to dark green, pale green beneath, glabrous, margin entire; base cordate, apex acute with acumen ca. 2 mm. ***Inflorescence*** ca. 2.5 × 0.5 cm, peduncle ca. 0.15 cm, with up to 8 flowers arranged in a one-sided spiral, one flower open at a time. ***Fertile bracts*** linear narrowly ovate, ca. 1.6 × 0.2 cm, green, pale red at apex, outer and inner surfaces glabrous, almost translucent, margin entire, apex acute. ***Bracteole*** linear elliptic, ca. 1.7 × 0.2 cm, white, outer and inner surfaces glabrous, almost translucent, margin entire, apex acute. ***Flower*** white, born singly from each bract, calyx 0.4 cm long, tubular, white, glabrous, corolla tube white, glabrous, dorsal lobe narrowly elliptic, ca. 0.7 × 0.2 cm, slightly concave, lateral lobes elliptic, ca. 0.7 × 0.1 cm, labellum obovate, ca. 0.7 cm × 0.4 cm, very light yellow at base, darker yellow spreading towards lip, curved forward, lateral staminodes elliptic, ca. 0.5 × 0.17 cm, white, apex acute, glabrous, stamen ca. 0.75 cm long; filament ca. 4 × 1 mm (widest at base), glabrous adaxially and abaxially, anther ca. 3.5 mm long, glabrous; anther crest rounded, glabrous; thecae oblong, ca. 0.1 × 0.05 cm, white, glabrous, dehiscing by pores, stigma cup-shaped, white, glabrous, epigynous glands 0.1 cm long, linear, apex pointed. ***Fruit*** not seen.

#### Distribution.

Endemic in Borneo, Sabah; known from Tawau and Tambunan.

#### Etymology.

The species is named after Mr. Linus Gokusing from Kipandi Park. He collected this species from Tawau in 2011. His passion for plants, such as orchids and gingers, has secured information useful to researchers, tourists and botany students.

#### Ecology.

Mix dipterocarp forest at 500–600 m elevation.

#### Conservation status.

**Figure 9.**
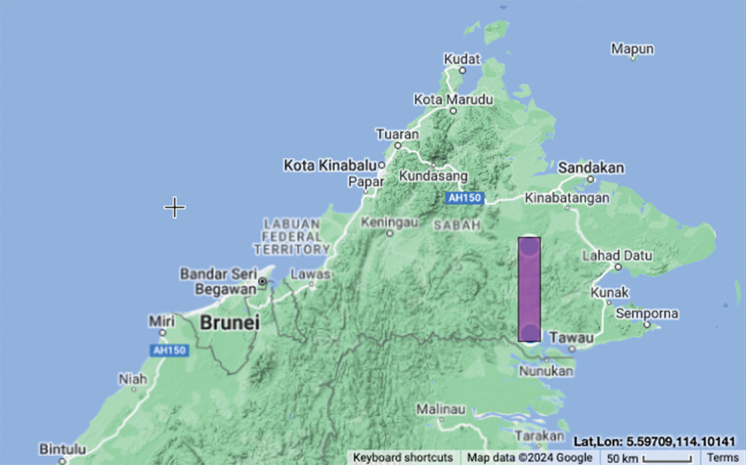
Distribution map of *Boesenbergiagokusingii*, EOO = 0 km^2^, AOO = 8 km^2^ (Bachman et al. 2011).

Vulnerable (VU D2). The species has only been documented at Tawau and Tambunan, Sabah, Malaysia, where only 3–6 populations were found at each site (Fig. 9).

## Supplementary Material

XML Treatment for Boesenbergia bosuangii

XML Treatment for Boesenbergia ganaensis

XML Treatment for Boesenbergia gokusingii

## ﻿Additional information

### Author ORCIDs

Nyee Fan Lam https://orcid.org/0000-0002-6371-4102

Axel Dalberg Poulsen https://orcid.org/0000-0002-7651-6439

### Data availability

The data underpinning the analysis reported in this paper are deposited at GBIF, the Global Biodiversity Information Facility, and are available at https://doi.org/10.15468/gm82es.
